# A study about the costoclavicular space in patients with pectus excavatum

**DOI:** 10.1186/s13019-014-0189-2

**Published:** 2014-12-06

**Authors:** Jae-Jun Kim, Hyung Joo Park, Jae Kil Park, Deog Gon Cho, Seok Whan Moon

**Affiliations:** Department of Thoracic and Cardiovascular Surgery, Uijeongbu St. Mary’s Hospital, The Catholic University of Korea College of Medicine, Seoul, South Korea; Department of Thoracic and Cardiovascular Surgery, Seoul St. Mary’s Hospital, The Catholic University of Korea College of Medicine, 222, Banpo-Daero, Seocho-gu, 137-701 Seoul South Korea; Department of Thoracic and Cardiovascular Surgery, St. Vincent’s Hospital, The Catholic University of Korea College of Medicine, Seoul, South Korea; Department of Thoracic and Cardiovascular Surgery, St. Paul’s Hospital, The Catholic University of Korea College of Medicine, Seoul, South Korea

**Keywords:** Pectus excavatum, Nuss procedure, Thoracic outlet syndrome, Costoclavicular space

## Abstract

**Background:**

The aim of the present study is to investigate the costoclavicular space in patients with pectus excavatum.

**Materials and methods:**

Between April and November 2011, consecutive 50 patients with pectus excavatum and consecutive 50 patients without pectus excavatum were included into the present study. The costoclavicular measurements (the shortest distance, the crossing angle) were measured for the costoclavicular investigation.

**Results:**

Firstly, there were no significant differences of the costoclavicular measurements in each and between symmetric and asymmetric subgroup, and in the overall, bilaterally. The shortest distance had a significant positive correlation with BMI (right p = 0.001, left p = 0.032) and a significant negative correlation with the crossing angle (right p = 0.013, left p = 0.001). Secondly, in the control group, the shortest distance had significant positive correlations with body weight and BMI (Body weight right p = 0.001, left p < 0.001; BMI right p = 0.001, left p < 0.001), and significant negative correlations with the crossing angles (right p = 0.002, left p < 0.001) and the sternal angle (right p = 0.032, left p = 0.017). Thirdly, the control group had the significant longer shortest distance than the pectus excavatum group (right p <0.001, left p <0.001). Fourthly, a decrease of the shortest distance (right p <0.001, left p <0.001), an increase of the crossing angle (right p < 0.001, left p < 0.001) and the sternal angle (p <0.001), and also a decrease of the Haller index (p <0.001) was found postoperatively.

**Conclusion:**

Patients with pectus excavatum originally have narrower costoclavicular spaces than the normal control group, and the postoperative costoclavicular space are much narrower also.

**Electronic supplementary material:**

The online version of this article (doi:10.1186/s13019-014-0189-2) contains supplementary material, which is available to authorized users.

## Background

Pectus excavatum is the most common congenital chest wall deformity and is characterized by a depression of the sternum, ribs, and the adjacent costal cartilages [1]-[3]. Several methods for the correction of pectus excavatum have been performed in the meantime, and the Nuss procedure has become the procedure of choice because of its less invasiveness and remarkable results [2]. Despite its remarkable results, several complications have been reported, including cardiopulmonary injury, pericardial and pleural effusion, wound infection, and bar migration [4]. Besides these complications, thoracic outlet syndrome (TOS) after the Nuss procedure was recently reported [4],[5]. TOS is a syndrome which occurs due to the compression of the neurovascular bundle which is composed of the brachial plexus, and the subclavian artery, or the subclavian vein [6]-[8]. We assumed that there would be the bony structural changes in the thoracic outlet, or in other words the costoclavicular space after the Nuss procedure, since ribs and the sternum are elevated by the Nuss procedure. Therefore, we analyzed the postoperative changes of the costoclavicular space after the Nuss procedures. In addition, the differences of the costoclavicular spaces between the normal control and the pectus excavatum group were investigated.

## Materials and methods

### Comparisons between the pectus excavatum and the control group

Between April and November 2011, consecutive 50 patients with pectus excavatum who underwent a Nuss procedure as the patient group and consecutive 50 patients without pectus excavatum as the normal control group were included into the present study. The inclusion criteria for the pectus excavatum group were set as: age from 10 to 19 years old, the preoperative diagnosis, no combined other congenital anomaly or disease, no trauma or other disease in the chest wall, shoulder and neck, no previous chest surgery, and no TOS complication after the Nuss operation. The data of the control group were collected out of the patients who had visited our hospital for other reasons and who underwent a 3-dimensional (3D) chest CT during the same period. The inclusion criteria for the control group were set as: age from 10 to 19 years old, no detectable chest wall deformity, no trauma or disease in the chest wall, shoulder, and neck, and no previous chest surgery.

3D Chest CT scans (SIEMENS, SOMATOM Definition, sensation 64) were routinely taken twice for preoperative evaluations, checkup of postoperative complications, and better operative results (once on the day before and once on the third day after surgery). All chest CT scans (3 mm slice thickness) were taken in hyper-abducted arm positions during a full inhalation. All chest CT scans were proved by the Institutional Review Board.

**Figure 1:**
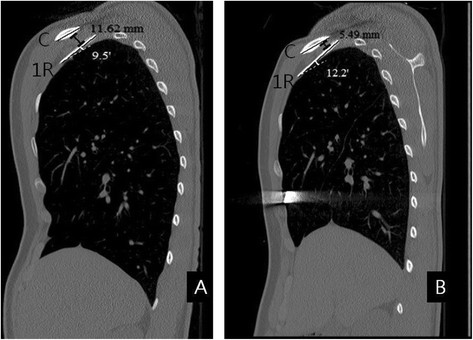
**Postoperative changes of the costoclavicular space in patient with pectus excavatum.** This patient was a 17- year- old male patient. The type of pectus excavatum was symmetric and the preoperative Haller index was 3.61. He underwent the Nuss procedure with a single metal bar. The shortest distance was defined as the shortest distance between the first rib and the clavicle on a sagital view of the 3-dimensional chest CT (black color). When the distance between the first rib and the clavicle was shortest, the angle between the long axis of the first rib and the clavicle was defined as the crossing angle (white color). Although the postoperative arm positions were more adducted than the preoperative arm positions bilaterally, the shortest distance became decreased and the crossing angle became increased, postoperatively (Figure **A**: preoperative status. Figure **B**: postoperative status, C: the clavicle, 1R: the first rib).

To investigate the costoclavicular space was measured the costoclavicular measurements (the shortest distance and the crossing angle) between the first rib and the clavicle on a sagital view of the 3D chest CT. All images were evaluated by the authors blindly, and image evaluations were performed on desktop computers installed with image measurement tools. We measured the minimum distance between the lower border of the clavicle and the upper border of the first rib clavicle on a sagital view of the 3D chest CT. The minimum distance was defined as the shortest distance. The angle between the long axis of the first rib and the clavicle was defined as the crossing angle when the distance between the first rib and the clavicle was shortest (Figure 1).

In the pectus excavatum group, the costoclavicular measurements were analyzed regarding the asymmetry and the severity of pectus excavatum. The severity of pectus excavatum was defined as the Haller index. The costoclavicular measurements were also analyzed in the control group. The costoclavicular measurements and the sternal angle between the pectus excavatum and the control group were compared. The angle between the manubrium of the sternum and the floor during chest CT scan was defined as the sternal angle. Age, height, body weight, body mass index (BMI), arm positions, severity, asymmetry, and the sternal angle were included into the parameters which influence the costoclavicular space. Arm positions were defined as the angles of abduction (i.e. the angles between the midline of the humerus and the median line of the thoracic spinal column on 3D chest CT). All analyses were performed bilaterally.

### Analyses of the postoperative changes of the costoclavicular space

The Nuss procedure was performed using one to two Nuss bars. When the parallel bar technique (two Nuss bars) was used, the upper Nuss bar was inserted into the 4th or 5th intercostal space and the lower Nuss bar was inserted into the 6th or 7th intercostal space.

The postoperative changes of the costoclavicular measurements and the sternal angle were analyzed, and the associations among the Haller index, the costoclavicular measurements changes, and the sternal angle changes after the Nuss procedure were also analyzed. In addition, the postoperative changes regarding the asymmetry and the number of the Nuss bar used for the Nuss procedure were analyzed.

### Statistical analysis

The comparisons between each group were analyzed with independent T or paired *T* test, when appropriate. Associated studies were evaluated using a Pearson correlation or a partial correlation analysis. The results were analyzed using the Statistical Package of Social Sciences (SPSS) computer software program, version 18.0 (Chicago, IL, USA). A p- value < 0.05 was considered to be statistically significant.

The present study was approved by the Institutional Review Board for Seoul St’ Mary Hospital (KC12RIS0809).

## Results

### Comparisons between the pectus excavatum and the control group

The pectus excavatum and the control group consisted of each 50 subjects. The basic clinical characteristics of each group are summarized in Table 1. Age and height between the two groups were not significantly different, but body weight and BMI of the control group were significantly larger than of the pectus excavatum group (Body weight p = 0.002, BMI p < 0.001). However, laboratory findings and physical examinations show no difference in nutrition status between them.

**Table 1:** **Basic clinical characteristics of the control and the pectus excavatum group**

	Group				P-value
	Pectus excavatum (n = 50)		Control (n = 50)		
	Mean	SD	Mean	SD	
Age (year)	14.22	2.51	14.98	2.38	0.123
Body weight (kg)	47.76	12.24	57.87	17.75	0.002
Height (m)	1.65	0.10	1.64	0.14	0.639
BMI (kg/m^2^)	17.69	2.45	21.18	4.86	<0.001

#### Pectus excavatum group

There was no significant difference in the arm positions and the costoclavicular measurements bilaterally in the overall pectus excavatum group. Association analyses showed the shortest distance had a significant correlation with BMI out of the influencing parameters bilaterally in the overall pectus excavatum group (right p = 0.001, left p = 0.032), and the shortest distances had a significant negative correlation with the crossing angle bilaterally in the overall pectus excavatum group (right p = 0.013, left p = 0.001). However, there was no significant association between the severity defined as the Haller index and the costoclavicular measurements bilaterally in the overall pectus excavatum group.

The symmetric and asymmetric types of the pectus excavatum group were 22 and 28, respectively. There was no significant difference in the arm positions bilaterally in each subgroup and between symmetric and asymmetric subgroup. Although all of the chest wall depressions in the asymmetric group were located at the right side, there was no significant difference in the costoclavicular measurements bilaterally in each, and between symmetric and asymmetric subgroup. The analyses of the costoclavicular measurements in the pectus excavatum group are summarized in Table 2.

**Table 2:** **Analyses of the costoclavicular measurements in the pectus excavatum group**

	Subgroups								
	Symmetric (n = 22)			Asymmetric (n = 28)			Total (N = 50)		
	Mean	SD	p-value	Mean	SD	p-value	Mean	SD	p-value
Shortest distance (mm)			0.764			0.645			0.888
Right	6.68	2.16		5.94	2.32		6.26	2.26	
Left	6.87	2.07		5.68	1.83		6.20	2.01	
Crossing angle (°)			0.621			0.152			0.523
Right	14.70	7.77.		13.93	6.02		14.27	6.78	
Left	13.56	7.42		16.42	6.77		15.16	7.14	

#### Control group

There was no significant difference in the arm position and the costoclavicular measurements bilaterally. Association analyses showed the shortest distance of the control group had significant positive correlations with body weight, BMI out of the influencing parameters bilaterally (Body weight right p = 0.001, left p < 0.001; BMI right p = 0.001, left p < 0.001), and the shortest distances had significant negative correlations with the crossing angles (right p = 0.002, left p < 0.001) and the sternal angle (right p = 0.032, left p = 0.017). The analyses of the costoclavicular measurements in the control group are summarized in Table 3.

**Table 3:** **Analyses of the costoclavicular measurements in the control group**

	Mean	SD	95% confidence interval	p-value
Difference (mm)			−0.450 – 2.620	0.164
Right shortest distance	9.83	4.49		
Left shortest distance	8.74	3.13		
Difference (°)			−2.180 – 3.928	0.571
Right crossing angle	13.55	7.54		
Left crossing angle	12.68	7.85		

#### The comparisons between the pectus excavatum and the control group

There were no significant differences in the arm positions between the pectus excavatum and the control group, bilaterally. The control group had the significant longer shortest distance than the pectus excavatum group (right p <0.001, left p <0.001), but there were no significant differences in the crossing angles between the two groups, bilaterally. Interestingly, there was no significant difference in the sternal angle between the pectus excavatum and the control group. The comparisons of the costoclavicular measurements and the sternal angle between the pectus excavatum and the control group are summarized in Table 4.

**Table 4:** **Comparisons of the costoclavicular measurements and the sternal angle between the pectus excavatum and the control group**

	Differences (pectus excavatum group– control group)			p-value
	Mean	SE	95% confidence interval	
Right shortest distance (mm)	−3.56	0.71	−4.977 – −2.146	< 0.001
Left shortest distance (mm)	−2.54	0.53	−3.583 – −1.492	<0.001
Right crossing angle (°)	0.71	1.43	−2.131 – 3.559	0.620
Left crossing angle (°)	2.48	1.50	−0.498 – 5.458	0.102
Sternal angle (°)	−9.10	7.50	−24.15 – 5.95	0.230

### Postoperative changes of the costoclavicular measurements in the pectus excavatum group

There were no significant differences in the postoperative arm positions and in the postoperative costoclavicular measurements, bilaterally. However, the postoperative arm positions were more adducted than the preoperative arm positions bilaterally (right p <0.001, left p <0.001). There were significant postoperative changes in the costoclavicular measurements, the sternal angle, and the Haller index. However, there were no significant associations among the Haller index changes, the costoclavicular measurements changes, and the sternal angle changes bilaterally after the Nuss procedure. The postoperative changes of the costoclavicular measurements, the Haller index, and the sternal angle in the pectus excavatum group are summarized in Table 5.

**Table 5:** **Postoperative changes of the costoclavicular measurements, the Haller index, and the sternal angle in pectus excavatum group**

	Postoperative paired differences (preoperative – postoperative value)			p-value
	Mean	SE	95% confidence interval	
Right shortest distance (mm)	1.84	1.71	1.392 – 2.293	<0.001
Left shortest distance (mm)	1.41	1.56	1.002 – 1.822	<0.001
Right crossing angle (°)	−2.78	6.99	−4.806 – −0.749	0.008
Left crossing angle (°)	−4.11	6.91	−6.119 – −2.106	<0.001
Sternal angle (°)	−4.648	0.97	−2.701 – − 6.597	<0.001
Haller index	1.33	0.91	1.070 – 1.593	<0.001

In addition, the costoclavicular measurements, the Haller index, and the sternal angle were analyzed regarding the asymmetry and the number of bar used for Nuss procedure. There were significant differences only in the postoperative Haller index regarding the asymmetry and the asymmetry group had the significantly larger postoperative Haller index than the symmetry group (p = 0.031). 38 of 50 patients (27 of 28 asymmetry patients and 11 of 22 symmetric patients) underwent the parallel bar technique (two Nuss bars) to correct the deformity of pectus excavatum. There were significant differences in the postoperative right crossing angle and the postoperative Haller index regarding the number of bar used for Nuss procedure. The right crossing angle in the two bars group were significantly smaller than of the one bar group (p = 0.022), and the postoperative Haller index in the two bars group was significantly larger than in the one bar group (p = 0.020). However, due to the small sample size, the costoclavicular measurements could not be fully analyzed regarding the level of the upper bar.

## Discussion

TOS is a classical compression result with three specific levels which are the interscalene triangle, the costoclavicular space and the coracopectoral tunnel [8]-[10]. Given that TOS after the Nuss procedure is extremely unusual, and the multifactorial issues which has been attributed to TOS, a study for TOS should include these multifactorial issues. However, we also experienced one case of TOS after the Nuss procedure during the same period and we found out that the costoclavicular space of the patient became much narrower postoperatively on the chest CT scan. Therefore, we assumed that there would be the bony structural changes in the thoracic outlet when the sternum, ribs and costal cartilages are elevated by the Nuss procedure and we investigated the costoclavicular space out of these three anatomic levels to evaluate the changes of the thoracic outlet after the Nuss procedure. In addition, the differences of the costoclavicular spaces between the normal control and the pectus excavatum group were investigated to find out the characteristics of the thoracic outlet in patients with pectus excavatum.

According to previous studies, the costoclavicular space can be changed by various factors, especially by arm positions [6],[7],[9]. As the arm position is more abducted, the costoclavicular space is narrower [6],[7],[9]. All the chest CT scans were taken in hyper-abducted arm positions and the present study results showed no significant differences in the arm positions in each subgroup and between the pectus excavatum and the control group, bilaterally also. So the statistics of these data could be easy analyzed.

In regards to the influencing parameters, the costoclavicular measurements in the control group showed significant associations with body weight and BMI. However, there was a significant association with only BMI in the pectus excavatum group. As mentioned above, these were the results of a smaller body weight and BMI in the pectus excavatum compared with the control group. With these results the correlation between the costoclavicular measurements and the body size (body weight and BMI) could be proved.

Although all the chest wall depressions were located at the right side in the asymmetric subgroup, the present study showed no significant differences in the costoclavicular measurements bilaterally in each subgroup and between the symmetric and the asymmetric subgroup. The control group had the significantly longer shortest distance compared with the pectus excavatum group bilaterally. In addition, there was no significant difference in the sternal angle between the pectus excavatum and the control group, and the severity and the sternal angle did not influence the costoclavicular measurements in the pectus excavatum group. With these findings, it could be assumed, that the differences in the costoclavicular measurements between the control and the pectus excavatum group did not result from the chest wall depression but from the overall variances or deformities of the skeletal system in the pectus excavatum group.

The present study showed a significant decrease of the shortest distance and a significant increase of the crossing angle bilaterally after the Nuss procedure. There was also a significant increase of the sternal angle and a significant decrease of the Haller index postoperatively. However, there were no significant associations among the Haller index changes, the costoclavicular measurements changes, and the sternal angle changes bilaterally after the Nuss procedure. With these findings, it could be assumed, that there were no significant associations among them, because there were the whole chest wall anatomical changes after the Nuss procedure and no useful index indicating the severity of the whole chest wall depression. In addition, the present authors suppose that the open reconstruction for the pectus excavatum will not lead the same impacts on the costoclavicular space, since the open reconstruction does not elevate the whole chest wall including the sternum, ribs, and costal cartilages. The further studies about TOS after the Nuss procedure will also influence the decision making process on the surgical methods for repair (Nuss procedure vs. open reconstruction).

There was a significant difference in the measurements between the one bar and the two bar group. And there is reported that after removing the uppermost metal bar to expand the possibly compressed the thoracic outlet, the rapid improvement in the vascular and neuronal symptoms [4]. This finding suggests that the uppermost bar could influence the costoclavicular measurements. However, due to the small sample size, the costoclavicular measurements could not be fully analyzed regarding the level of the upper bar. The study about the influences of the level of upper bar on the costoclavicular space is required.

The present study showed the postoperative changes and the anatomic differences of the costoclavicular space between the pectus excavatum and the normal control group. However, the real mechanism of the postoperative costoclavicular space changes remains still unclear.

## Conclusion

Patients with pectus excavatum originally have narrower costoclavicular spaces than the normal control group, and the postoperative costoclavicular space in patients with pectus excavatum are much narrower also. The further studies on the costoclavicular space including the mechanism of postoperative changes will be needed, and these studies will provide a deeper and wider comprehension of pectus excavatum.

### Consents

Written informed consent was obtained from the patient for the publication of this report and any accompanying images.

## Authors’ original submitted files for images

Below are the links to the authors’ original submitted files for images. Authors’ original file for figure 1

## Abbreviation

TOS: Thoracic outlet syndrome
