# Supplementary figures and images for: A study about the costoclavicular space in patients with pectus excavatum
Source: J Cardiothorac Surg. 2014 Dec 6;9:189. doi: 10.1186/s13019-014-0189-2 (PMC4266879; doi:10.1186/s13019-014-0189-2)

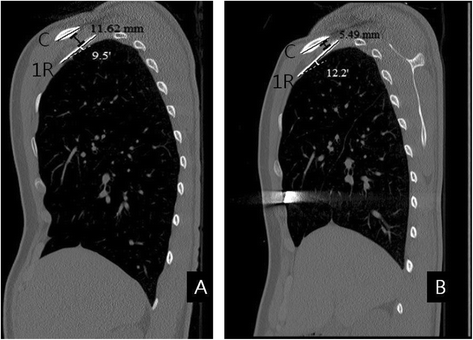

Supplement: Supplementary file 1 — Authors’ original file for figure 1 [file 13019_2014_189_MOESM1_ESM.gif]
